# Facilitators and barriers to building research capacity among clinical nurses: A systematic review protocol

**DOI:** 10.1371/journal.pone.0331292

**Published:** 2025-08-29

**Authors:** Jinglin Song, Yunhong Lei, Luhuan Yang, Jordan Tovera Salvador, Daying Long

**Affiliations:** 1 School of Nursing, Philippine Women’s University, Manila, Philippines; 2 Yichang Hubo Medical Research Institute, Yichang City, Hubei Province, China; 3 Academic Research Center, Yichang Hubo Medical Research Institute, Yichang City, Hubei Province, China; 4 Department of Nursing Education, College of Nursing, Imam Abdulrahman Bin Faisal University, Dammam City, Eastern Province, Saudi Arabia; 5 The Department of Hepatobiliary, Pancreatic and Splenic Surgery, The central hospital of Enshi Tujia and Miao Autonomous Prefecture (Enshi Clinical College of Wuhan University), Enshi City, Hubei Province, China; Alexandria University Faculty of Nursing, EGYPT

## Abstract

**Background:**

Clinical nurses’ research capacity is essential for building a research-active nursing workforce and advancing evidence-based practice. Despite increasing global attention, few systematic reviews have examined the current state of nurses’ research capacity or the factors influencing its development.

**Objective:**

This protocol outlines a systematic review designed to evaluate clinical nurses’ research capacity and to identify the key barriers and facilitators to its improvement across diverse healthcare contexts.

**Methods:**

The review will follow PRISMA guidelines. Eight databases—PubMed, Cochrane Library, Embase, CINAHL, Web of Science, Scopus, CNKI, and Wanfang—will be searched for relevant quantitative, qualitative, and mixed-methods studies. Study selection, data extraction, and quality appraisal will be performed independently by two reviewers using the Mixed Methods Appraisal Tool (MMAT). Findings will be integrated through thematic synthesis based on Braun and Clarke’s six-phase framework. Quantitative results will be transformed into narrative form and synthesized with qualitative data. Given the anticipated heterogeneity, a meta-analysis will not be conducted.

**Conclusion:**

The findings are expected to provide insights into clinical nurses’ research capacity and its influencing factors to inform targeted strategies to support research engagement and integration into clinical practice.

## Introduction

Clinical nursing practice is increasingly guided by evidence-based decision-making, which depends on nurses’ research capacity [1–3]. Research capacity equips clinical nurses with the knowledge and skills to apply evidence in practice. This, in turn, helps improve patient outcomes and advance the nursing profession [1,4–7]. International organizations such as the International Council of Nurses (ICN) and the Nursing and Midwifery Council (NMC) emphasize enhancing nursing research capacity through policy, education, and professional standards [8,9]. Registered nurses (RNs) are expected to have the ability to think critically, apply their knowledge and skills effectively, and deliver expert, evidence-informed care [9,10].

Despite these expectations, studies from various countries consistently report low levels of research capacity among clinical nurses [11–13]. For example, 77% of nurses in a Norwegian hospital rated their research competence as weak or only acceptable [14]. Similarly, a survey in Western Australia found low engagement in research activities [15]. In China, clinical nurses—particularly those in primary hospitals—exhibit limited understanding and capacity in research [16,17]. These deficits hinder the implementation of evidence-based interventions and restrict both care quality and professional growth [18,19].

Several factors contribute to this gap. Clinical nurses often encounter substantial barriers such as heavy workloads and time constraints, which significantly limit their capacity to engage in research [20–22]. Inadequate training and lack of support further diminish nurses’ confidence and motivation to engage in research [23].

Recent studies have increasingly focused on clinical nurses’ research capacity, identifying various environmental and individual factors that influence their research abilities [24,25]. However, these studies are often fragmented and lack systematic integration and rigorous analysis. To date, no comprehensive review has systematically assessed the current state of clinical nurses’ research capacity or consolidated the facilitators and barriers influencing its development.

This systematic review aims to address this gap by (1) evaluating the current level of research capacity among clinical nurses, and (2) identifying the key factors—both barriers and enablers—that affect its development.

### Definition of research capacity

Based on prior concept analysis in the literature, research capacity is conceptually defined as the ability to sustainably conduct nursing research within a specific context [1]. Operationally, research capacity refers to clinical nurses’ ability to apply research knowledge and skills in the context of their clinical work. This includes key research activities such as identifying clinical problems, reviewing literature, and disseminating findings—areas further detailed in the five core dimensions listed in Table 1. This capacity can be assessed through self-reported questionnaires, evaluations by nursing managers, and objective indicators of research output, such as participation in research projects, peer-reviewed publications, and conference presentations.

**Table 1:** Core dimensions for assessing clinical nurses’ research capacity.

*Dimension No.*	*Dimension Name*
*1*	*Problem identification and literature searching*
*2*	*Research design and ethics*
*3*	*Data collection and analysis*
*4*	*Research writing and communication*
*5*	*Attitudes and behavioral tendencies*

## Methods

### Study registration and review timeline

The study protocol has been registered with the International Platform for Prospective Registration of Systematic Reviews (PROSPERO) (CRD42024594322). This study will follow the Preferred Reporting Items for Systematic Reviews and Meta-analysis Protocols (PRISMA-P) list (see S1 File) [26].

The estimated duration for each review stage is as follows: literature search (2 weeks), title and abstract screening (2 weeks), risk of bias assessment (1 week), full-text review and data extraction (4 weeks), data analysis and synthesis (4 weeks), and manuscript drafting and revision (8 weeks). The entire review is expected to be completed within approximately six months.

### Review questions

1. What is the current level of research capacity among clinical nurses?
2. What are the key barriers and facilitators influencing clinical nurses’ research capacity?

The questions were developed based on the PICo framework (Population, Phenomenon of Interest, Context), as presented in Table 2.

**Table 2:** Research questions structured using the PICo framework.

*PICo element*	*Description*
*P (Population)*	*Clinical nurses.*
*I (Phenomenon of Interest)*	*Level of research capacity and associated influencing factors.*
*Co (Context)*	*Clinical practice within hospital wards.*

### Eligibility criteria

#### Inclusion criteria.

Studies will be included if they meet the following criteria: (a) Population: Clinical nurses; (b) Phenomenon of Interest: The study explores research capacity and/or its associated factors, such as barriers and facilitators; (c) Context: Clinical practice settings in hospitals; (d) Study design: Peer-reviewed quantitative, qualitative, or mixed-methods studies; (e) Language and search period: Published in English or Chinese from the inception of the database to July 2025.

#### Exclusion criteria.

Studies will be excluded if they meet any of the following conditions: (a) Incomplete or missing data that cannot be retrieved after contacting the corresponding author; (b) Duplicate publications or overlapping data reported by the same author or research team; (c) Non-original research articles, including reviews, letters, books, conference abstracts, or guidelines; (d) Full text not available.

### Search strategy

Eight databases—PubMed, Cochrane Library, Embase, CINAHL, Web of Science, Scopus, CNKI, and Wanfang—will be searched for English- and Chinese-language studies on clinical nurses’ research capacity. The search strategy will combine MeSH terms and free-text keywords related to clinical nurses, research capacity, and associated factors, using Boolean operators and adapted to each database’s syntax. The search will cover studies from database inception to July 2025. A sample PubMed search strategy is presented in Table 3.

**Table 3:** Search strategy in PubMed.

*Database*	*Medline*
*Platform*	*Pubmed*
*Search*	*query*
*#1*	(“clinical nurse*”[Title/Abstract] OR “registered nurse*”[Title/Abstract] OR “staff nurse”[Title/Abstract] OR “nursing staff”[Title/Abstract] OR “professional nurse*”[Title/Abstract] OR “nurse practitioner*”[Title/Abstract] OR “nurse*”[Title/Abstract] OR “advanced nurse*”[Title/Abstract] OR “specialist nurse*”[Title/Abstract] OR “nurse specialist”[Title/Abstract]) OR (nurses[MeSH Terms])
*#2*	“research capacity”[Title/Abstract] OR “research capability”[Title/Abstract] OR “research competence”[Title/Abstract] OR “research ability”[Title/Abstract] OR “research skill*”[Title/Abstract] OR “research proficiency”[Title/Abstract]
*#3*	“influencing factor*”[Title/Abstract] OR “facilitators and barriers”[Title/Abstract] OR “hindrance factor*”[Title/Abstract] OR “predictor*”[Title/Abstract] OR “related factors”[Title/Abstract] OR “promotive factor*”[Title/Abstract] OR “risk factor*”[Title/Abstract]
*#4*	*#1 AND #2 AND #3*

* Indicates a wildcard to identify terms that begin with the given string.

### Data selection

All retrieved records will be imported into Covidence for management and screening, where duplicates will be removed. To minimize selection bias, two independent reviewers will screen the titles and abstracts. Discrepancies will be resolved through discussion, and if consensus cannot be reached, a third reviewer will make the final decision. The same two reviewers will then independently screen the full texts of potentially eligible studies, with disagreements resolved in the same manner.

### Data extraction

A standardized electronic data extraction form will be developed based on the objectives of this review, key variables identified in the prior literature, and recommendations from the Joanna Briggs Institute (JBI) guidelines [27]. The form will be pilot-tested on three purposively selected eligible studies representing different study designs. Feedback from the pilot testing will be incorporated to refine the extraction form prior to commencing formal data extraction. Two reviewers will then independently extract data using the finalized form. Disagreements between reviewers will be addressed through discussion, and if unresolved, adjudicated by a third reviewer.

Based on the current review objectives, the form is expected to collect the following information from each included study: the basic study information (authors, country, and publication year), study design, population characteristics, research methods (study sample, measurement tools, and analysis methods), main findings, limitations, conclusions, and study quality.

Barriers are defined as factors impeding clinical nurses’ development or application of research capacity (e.g., time constraints, insufficient staffing, lack of training or mentorship). Facilitators, in contrast, are factors enhancing research engagement (e.g., institutional support, training programs, protected research time). These factors will be extracted based on author-reported classifications. Where absent, an inductive coding approach guided by Braun and Clarke’s thematic analysis will be used, with categorizations developed through team consensus [28].

### Risk of bias assessment

The methodological quality and risk of bias of the included studies will be evaluated using the Mixed Methods Appraisal Tool (MMAT, 2018 version) [29]. The MMAT is specifically designed to appraise qualitative, quantitative, and mixed-methods studies. It assesses key quality criteria, including: (1) the clarity and relevance of the research aim; (2) alignment between research questions and data sources; (3) appropriateness of the study design and methodology; (4) adequacy of the sampling strategy; (5) rigor in data collection and analysis; (6) clarity in presenting results; and (7) coherence between findings and conclusions. For mixed-methods studies, the integration of qualitative and quantitative components is also examined.

Two reviewers will independently perform the quality appraisal. Any discrepancies will be resolved through discussion, or by consulting a third reviewer when necessary, to ensure the reliability and objectivity of the assessment process. The results of the quality assessment will inform the interpretation of the review findings and be reported in a summary table.

### Data synthesis

Articles meeting the inclusion criteria will be categorized according to their methodological design (e.g., quantitative studies,qualitative, or mixed-methods studies). Data will be systematically extracted into standardized tables using a predefined data extraction form. Extracted variables will include study characteristics (e.g., author, year, country), sample and setting details, instruments used to assess research capacity, and reported outcomes, including associated barriers and facilitators.

Two reviewers will independently conduct a thematic synthesis of all included studies using NVivo (version 12), following Braun and Clarke’s six-phase framework [28]. To enable integration, quantitative findings will be transformed into textual descriptions (i.e., qualitized) and synthesized with qualitative data. For mixed-methods studies, qualitative and quantitative components will be analyzed separately and then integrated into the overall thematic synthesis.

A meta-analysis will not be feasible because of the expected methodological and contextual heterogeneity, including variations in study design, measurement tools, populations, and outcomes. Themes and subthemes will be generated through an iterative, reflexive process. This will involve repeated review of extracted data, continuous cross-study comparison, and refinement through multiple team meetings. Preliminary themes will be checked against the original data for accuracy and conceptual coherence. Final themes will be confirmed through collaborative team discussion and, when necessary, validated by an external researcher with subject expertise. An audit trail documenting coding decisions and theme development will be maintained to ensure transparency and trustworthiness.

### Limitations

Despite the strengths of this protocol, several limitations should be acknowledged. First, due to resource constraints, only studies published in English or Chinese will be included. This language restriction may introduce publication and language bias, potentially limiting the comprehensiveness and global applicability of the findings. Second, the study population is limited to clinical nurses working in hospital settings, which may affect the generalizability of the results to nurses in non-clinical, administrative, community, or specialized roles. Third, while qualitative, quantitative, and mixed-methods studies will be included, methodological heterogeneity—such as differences in study design, measurement instruments, and healthcare contexts—may challenge data comparability and synthesis. Finally, grey literature (e.g., dissertations, theses, and unpublished reports) will be excluded, which may further increase the risk of publication bias.

## Discussion

Globally, there is a growing emphasis on enhancing clinical nurses’ research capacity to facilitate the integration of research into practice. For example, O’Byrne and Smith reviewed international models for developing clinical nurses’ research capacity. They emphasized the importance of organizational support, mentorship, and protected research time for embedding evidence-based practice in clinical care [30]. Strengthening research capacity is vital for cultivating a research-active workforce and promoting the generation and application of evidence in daily clinical decision-making.

This protocol presents a systematic framework for evaluating the current state of clinical nurses’ research capacity and identifying key barriers and facilitators that shape its development. While the specific influencing factors will be derived from the data during the review process, the use of Braun and Clarke’s six-phase reflexive thematic analysis will ensure a transparent and rigorous approach to synthesizing findings across diverse study designs. Influencing factors will be extracted and categorized thematically, allowing for a structured understanding of how research capacity is developed, supported, or constrained across settings.

The anticipated findings will help identify priority areas for future research and inform the development of targeted interventions. For example, studies have consistently reported that nurses often lack formal training in research methods and scientific writing, which limits their capacity to engage in independent or collaborative research [ 31,32]. Moreover, initiatives such as the Nurse Scholars Program described by Chlan et al. demonstrated how structured mentorship and protected research time significantly increased research engagement among clinical nurses in a large U.S. hospital [33]. These examples underscore actionable areas for system-level interventions, such as competency-based training programs, sustainable mentorship models, and institutional policies that allocate dedicated time for research.

By mapping the existing literature and synthesizing evidence across different contexts, this review aims to generate practical, evidence-informed recommendations for healthcare leaders, educators, and policymakers. Ultimately, the findings will contribute to building a more research-capable nursing workforce, facilitating the integration of research into clinical practice, and improving the quality and effectiveness of patient care.

## Supporting information

S1 File PRISMA-P checklist. (DOCX)
